# Granulocyte-Colony Stimulating Factor Improves MDX Mouse Response to Peripheral Nerve Injury

**DOI:** 10.1371/journal.pone.0042803

**Published:** 2012-08-13

**Authors:** Gustavo Ferreira Simões, Alexandre Leite Rodrigues de Oliveira

**Affiliations:** Department of Structural and Functional Biology, Institute of Biology, University of Campinas (UNICAMP), Campinas, Brazil; Universidad Federal de Santa Catarina, Brazil

## Abstract

**Background:**

G-CSF has been shown to increase neuronal survival, which may positively influence the spinal cord microenvironment during the course of muscular dystrophies.

**Methodology/Principal Findings:**

Male MDX mice that were six weeks of age received a left sciatic nerve transection and were treated with intraperitoneal injections of 200 µg/kg/day of G-CSF 7 days before and 7 days after the transection. The axotomy was performed after the cycles of muscular degeneration/regeneration, consistent with previous descriptions of this model of muscular dystrophy. C57BL/10 mice were used as control subjects. Seven days after the surgery, the animals were sacrificed and their lumbar spinal cords were processed for immunohistochemistry (anti-MHC I, anti-Synaptophysin, anti-GFAP and anti-IBA-1) and transmission electron microscopy. MHC I expression increased in both strains of mice after the axotomy. Nevertheless, the MDX mice displayed a significantly smaller MHC I upregulation than the control mice. Regarding GFAP expression, the MDX mice showed a stronger astrogliosis compared with the C57BL/10 mice across all groups. Both groups that were treated with G-CSF demonstrated preservation of synaptophysin expression compared with the untreated and placebo groups. The quantitative analysis of the ultrastructural level showed a preservation of the synaptic covering for the both groups that were treated with G-CSF and the axotomized groups showed a smaller loss of synaptic contact in relation to the treated groups after the lesion.

**Conclusions/Significance:**

The reduction of active inputs to the alpha-motoneurons and increased astrogliosis in the axotomized and control groups may be associated with the cycles of muscle degeneration/regeneration that occur postnatally. The G-CSF treated group showed a preservation of the spinal cord microenvironment after the lesion. Moreover, the increase of MHC I expression in the MDX mice that were treated with G-CSF may indicate that this drug performs an active role in regenerative potential after lesions.

## Introduction

The use of MDX mice is a well-established experimental model for studying Duchenne Muscular Dystrophy[1]–[8]. During the course of the disease, the MDX mice present degeneration/regeneration muscle cycles [3], [8], [9]. Studies have demonstrated that, on the first postnatal day, the MDX mice’s muscle fibers already show a discontinuation of sarcomeres and disorganized myofibrils, thus characterizing muscle degeneration processes [3]. In another study, the authors showed that two-week-old MDX mice showed signs of muscle degeneration/regeneration such that some of the muscle fibers displayed central nuclei and cellular infiltrates. These changes were widespread in most of the muscles during the animals’ third week of life [8]. The same authors have showed that, during the sixth week of life, 50% of all muscle fibers of the hind limbs presented central nuclei. This myonecrosis is often preceded by the collapse and detachment of the basal lamina of the sarcolemma and subsequent muscle fiber degeneration, and it is associated with extensive inflammation. During such a process, CD4+ and CD8+ accumulate in the MDX mice’s skeletal muscles, especially from the fourth postnatal week [10]. In addition, during muscle regeneration, B lymphocytes proliferate and the secretion of IFN-β increases.

Apart from the characteristic muscle degeneration, due to the absence of dystrophin, in MDX mice, the lack of such a protein in the central nervous system (CNS) has also been implicated in neuronal loss [11], [12]. In this sense, it has been suggested that dystrophin performs an important role in developing and maintaining the structural and functional properties of neurons [12]. Evidence of abnormal connections in the brains of adult MDX mice was first demonstrated by Carreta et al. [13]. Previously, it had been demonstrated that there was a decrease in the number of axons in the corticospinal tract of MDX mice [12]. This change indicated an important role for dystrophin in the cerebral cortex, and the complete loss of dystrophin’s expression in the CNS of the MDX mice also altered its neural function [14].

A previous study showed that MDX mice presented additional spinal cord alterations [15]. Synaptic changes in alpha-motoneurons during the course of the disease were described, especially a decrease in synaptic inputs and an increased glial reaction in the motor column. Associated with this, a decreased expression of the major histocompatibility complex of class I (MHC I) was detected in MDX mice. It has been shown that MHC I expression by neurons and glial cells is correlated with post-lesion synaptic plasticity and ultimately with the regenerative response following injury. Such changes were correlated with the course of the disease and were regarded as additional contributors to the deterioration of motor function and to the decrease in regenerative potential that follows a lesion [15].

To date, although there is no consensus regarding DMD treatment, glucocorticoid corticosteroid (prednisone and deflazacort) prescriptions have been used as standard therapies [16]. However, it is possible that neuroprotective pharmacological treatments may result in a positive response during the course of the disease, which also reduces the inflammatory response at the peripheral level. With respect to this possible positive response, granulocyte colony stimulating factor (G-CSF) has been shown to have neurotrophic action [17], [18], including anti-apoptoptic activity in neurons [19], [20], [21], [22], to stimulate neovascularization [23]–[26], to present an anti-inflammatory effect[26]–[30] and to stimulate neurogenesis [31], [32].

G-CSF is a glycoprotein that contains 19.6 kilo Daltons and is a member of the cytokine family of growth factors. G-CSF was initially described more than twenty years ago as an inducer of cell differentiation in leukemic monocytic WEHI-3B [34], [35]. G-CSF has received FDA (U.S. Food and Drug Administration) approval and is commonly used to treat neutropenia[35]–[37] or to enhance success in bone marrow transplants [34], [38]. The known sources of G-CSF are monocytes, mesothelial cells, fibroblasts and endothelial cells. G-CSF stimulates the growth of neutrophil precursors and critically regulates the survival of mature post-mitotic neutrophils by inhibiting apoptosis [34], [38], [39].

The administration of G-CSF mobilizes stem and progenitor cells from bone marrow into the circulatory system, which in turn cross the blood-brain barrier and head toward the affected area in the CNS. Thus, G-CSF promotes anti-apoptotic effects on alpha-motoneurons after sciatic nerve axotomy in neonatal mice [40]. Moreover, this treatment induces upregulation of G-CSF receptors in such neurons, which may improve the effectiveness of the drug [40]. Studies of spinal cord injuries have shown that G-CSF enhanced motor recovery[41]–[45] and increased the vascularization of the lesioned microenvironment [44], which preserved the microenvironment within the lesioned area. [46], [47].

Lee et al. [22] showed that, after ischemic injury in the CNS, G-CSF treatment increased the production of vascular endothelial cells and decreased the length of the ischemic area. Concomitantly, it induced neurological functional recovery by providing a neuroprotective effect that reduced the extent of infarction and inflammatory infiltration [22]. In another study, the authors demonstrated a reduction in the signs of the disease in mice that were induced to the experimental autoimmune encephalomyelitis (EAE) following treatment with G-CSF [48]. The effects promoted by G-CSF that should be highlighted [48] include the maintenance of the integrity of the myelin sheath of the axons that were located in the cerebellum [48], a reduction in the recruitment of T cells to the CNS [48], discrete signs of inflammation and a limiting of the production of TNF-α [48], which is associated with early infiltration and neurological deficits.

### Aims

Based on the literature noted above, the aim of this study was to investigate the possible neuroprotective effects of G-CSF on the synaptic elements that related to spinal alpha-motoneurons following an axotomy of the sciatic nerve in MDX mice. Additionally, the immunomodulatory action of G-CSF on the expression of MHC I by neurons and glial cells and on glial reactivity-related markers was studied using immunohistochemistry. The overall benefit of the treatment was evaluated by analyzing both the ultrastructure of the spinal cord and spinal motoneurons and the pre-synaptic inputs that were located opposite to the neuron cell body.

## Materials and Methods

### Experimental Animals

Six-week-old male (20–25 g) mice from the strains C57BL/10 and MDX were used. The mice were obtained from the Multidisciplinary Center for Biological Research (CEMIB) at the University of Campinas, Brazil. The animals were grouped in plastic cages and were permitted free access to food and water. The animals’ cages were maintained with a controlled light/dark cycle (12 h) and at a temperature of 21°C. The experiments were carried out in accordance with the ethical principles for animal experimentation adopted by the Brazilian College of Animal Experimentation (COBEA) and were approved by the Ethics Committee for Animal Experimentation (CEUA), protocol number 1776-1. The animals were divided into six groups (axotomized/ipsilateral+G-CSF–n = 10, axotomized/ipsilateral/non treated–n = 10, axotomized/ipsilateral+placebo–n = 5, axotomized/contralateral+G-CSF treated–n = 10, axotomized/contralateral/non treated–n = 10, axotomized/contralateral+ placebo–n = 5 and normal animals–n = 10). In the treated groups, 200 mg/kg/day of G-CSF was administered subcutaneously to the mice for seven days before and seven days after axotomy. In the placebo groups, the mice received subcutaneous injections of 200 µl of 5% glucose (G-CSF vehicle) seven days before and seven days after axotomy.

### Sciatic Nerve Injury

The mice were anesthetized with a mixture of Vetaset (ketamine, Fort Dodge, 50 mg/kg) and kensol (xylazine, Körnig, 10 mg/kg), totaling 0.12 ml/25 g of body weight. After trichotomy at the left mid-thigh, a skin incision approximately 1.5 cm long was made using a scalpel. The skin and the thigh muscles were carefully retracted, exposing the sciatic nerve, which was injured at the level of the obturator foramen. The sciatic nerve transection was performed with iris microscissors. A 2-mm segment of the distal stump of the nerve was removed and diverted from its natural direction to avoid realignment between the stumps. The muscles were repositioned, and the skin was sutured. After surgery, the mice were maintained in a vivarium at the Nerve Regeneration Laboratory, Department of Anatomy, Institute of Biology, Unicamp, until they were sacrificed. Following sacrifice, the spinal cords were obtained and processed for immunohistochemistry and transmission electron microscopy. After fixation, the specimens were dissected and kept in fixative for 12 hours at a temperature of 4°C.

### Immunohistochemistry

For immunohistochemistry, the lumbar spinal cords were embedded in Tissue-Tek (Miles Inc., USA) and frozen in liquid nitrogen at −40°C for cryostat sectioning (12 µm). Primary rabbit anti-synaptophysin (1∶100, Dako, Glostrup, Denmark), goat anti-glial fibrillary acidic protein (GFAP) (1∶100, Santa Cruz Biotechnology, Inc., Santa Cruz, CA, USA), rabbit anti-IBA-1 (1∶600, Wako, Chuo-Ku, Osaka, Japan) and rat anti-MHC class I (1∶100, BMA Biomedicals AG, Augst, Switzerland) anti-sera were used. The anti-sera were diluted in a solution that contained BSA and Triton X-100 in 0.01 M PBS. The sections were incubated overnight at 4°C in a moist chamber. After being rinsed in 0.01 M PBS, the sections were incubated with Cy3- or Cy2-conjugated secondary anti-sera (1∶250, Jackson Immunoresearch, West Grove, PA, USA) for 45 min in a moist chamber at room temperature. The sections were then rinsed in PBS, mounted in a mixture of glycerol/PBS (3∶1), and observed using a Nikon TS100 microscope that was equipped with a digital camera (Nikon, Tokyo, Japan, DXM1200i). For quantitative measurements, the lesioned segments were first identified by decreased synaptophysin immunolabeling in the motoneuron microenvironment, combined with an increased glial reaction. Three alternate sections (the ipsi and contralateral sides of the spinal cord) were chosen from each animal (*n = *5 for each group). These sections were used to capture images from the ventral horn at a final magnification of ×200 with all of the settings unchanged. Quantification was performed using the enhance contrast and density slicing feature of IMAGEJ software (version 1.33u, National Institutes of Health, USA). The integrated density in pixels was measured in eight areas per motoneuron present on the lateral motor nucleus from each side. Lesioned neurons were visualized by the decreased synaptophysin labeling around the cell body. The non lesioned/lesioned ratio of the integrated density of the pixels was calculated for each section (for the axotomy groups) and then as a mean value for each spinal cord. The data were represented as the mean ± standard error of the mean (se).

### Electron Microscopy

The lumbar spinal cords were dissected out and stored overnight in fixative (1.5% paraformaldehyde, 2.5% glutaraldehyde in 0.1 M PB, pH 7.34) at 4°C. The specimens were then trimmed, osmicated, dehydrated and embedded in Durcupan (Fluka, Steinheim, Switzerland). Ultrathin sections from the L4–L6 segments were placed on formvar-coated copper grids, contrasted with uranyl acetate and lead citrate, and examined under a Tecnai Biotwin G^2^ Spirit transmission electron microscope (FEI Company, The Netherlands), which was operated at 120 kV. Neurons that possessed large bodies (>35 µm in diameter), which were found in the sciatic motoneuron pool and cut in the nuclear plane, were identified as alpha-motoneurons by the presence of C-type nerve terminals. Neurons were identified as axotomized based on the occurrence of chromatolytic changes in the cell bodies. A qualitative analysis was performed for all of the experimental groups. The surfaces of the experimental animals’ cells were photographed at a magnification of 11,000× by an Eagle 2 K video camera (FEI Company, The Netherlands) that was connected to a computer system. The images were then mounted together using vector graphics software. The synaptic terminals that opposed the motor neuron somata were identified, and the membrane coverings of all of the terminals were measured and calculated as both a percent of the membrane length and their numbers per 100 µm of cell membrane. These measurements were carried out using the measurement tool of the Image Tool software (Version 3.0, The University of Texas, Health Center in Santo Antonio, USA). The terminals were typed according to the nomenclature as S-type (with spherical synaptic vesicles that contained glutamate as the neurotransmitter), F-type (with flattened synaptic vesicles alone or flattened and spherical vesicles that contained glycine/gamma-aminobutyric acid (GABA) as the neurotransmitter) or C-type (with a subsynaptic cistern that contained acetylcholine as the neurotransmitter) [49]. The distance between consecutive nerve terminals that covered the motoneurons was also determined. A total of 120 alpha-motoneurons were studied. Two motoneurons were analyzed from each nonlesioned mouse, totaling 40 nonlesioned neurons (*n = *20 for each strain–MDX and C57BL/10). Additionally, two motoneurons were sampled from each side of the spinal cord from the axotomized mice, totaling four motoneurons per animal (axotomized/untreated: *n* = 20– ipsilateral, *n* = 20– contralateral; axotomized+G-CSF: *n* = 20– ipsilateral, *n* = 20 contralateral).

### Statistical Analysis

The data are presented as the mean ± SEM and were analyzed using one-way ANOVA followed by *Bonferroni post hoc* test for multiple comparisons at *P*<0.05 (*), *P*<0.01(**) and *P*<0.001 (***).

## Results

### MHC I Expression after Treatment with G-CSF

No differences in MHC I expression were observed in the nonlesioned animals across the mice strains (Figure S1).

**Figure 1:**
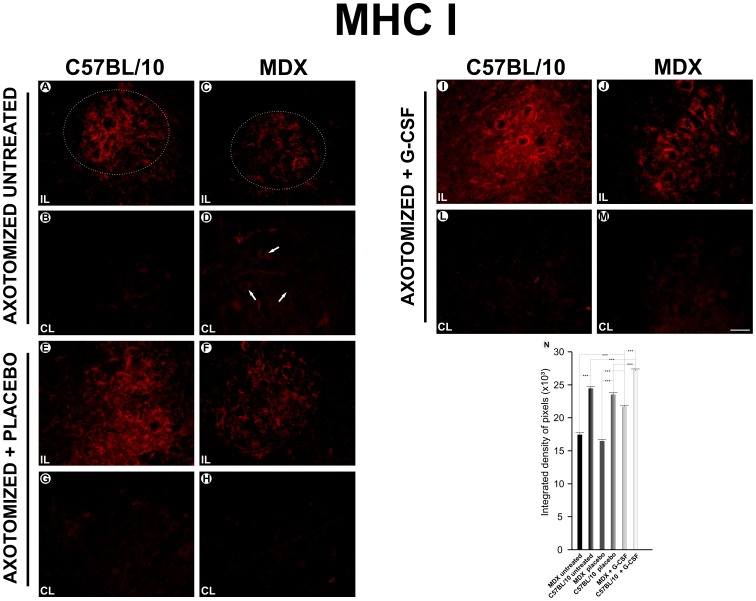
Anti-MHC I immunostaining one week after axotomy. **A** and **C**, the ipsilateral (IL) and contralateral ventral column of the spinal cord of the C57BL/10 strain seven days after axotomy; **B** and **D**, the ipsilateral and contralateral ventral column of the spinal cord of the MDX strain seven days after axotomy; **E** and **G**, the ipsilateral and contralateral of the C57BL/10 ipsilateral+ placebo group seven days after the axotomy; **F** and **H**, the ipsilateral and contralateral of the MDX ipsilateral+ placebo group; **I** and **L**, the ipsilateral and contralateral of the C57BL/10 ipsilateral+ G-CCSF group; **J** and **M**, the ipsilateral and contralateral of the MDX ipsilateral+ G-CSF group. (**N**) the quantitative analysis of the integrated density of the pixels between the left sides of the groups studied. In all experiments: n = 5. In **A–M**, magnification, *X200* (scale bar, 50 µm). The alpha-motoneurons were quantified in 3 distinct fields along the lumbar intumescence. In **N**, ***p<0.001 *vs*. CT, values are means ± SEM.

In the ipsilateral groups, increased immunoreactivity was observed in the region of the lateral motor nucleus in the ventral column of the spinal cord (Figure 1). The ipsilateral/untreated group showed a similar MHC I expression to the ipsilateral+placebo group. Overall, the C57BL/10 strain showed a greater MHC I expression in all experimental groups compared with the MDX animals. After treatment with G-CSF, a significant increase in the MHC I expression was observed in both strains, especially in the MDX mice (a 31% increase was observed compared with the placebo group and a 24% increase compared with the untreated group). The C57BL/10 G-CSF treated mice showed an MHC I expression increase of 15% compared with the placebo group and an 11% increase compared with the untreated group. All of the quantitative data that were described in this section are presented as supporting information in Table S1.

### Immunoreactivity Against Synaptophysin Following Sciatic Nerve Axotomy and Treatment with G-CSF

**Figure 2:**
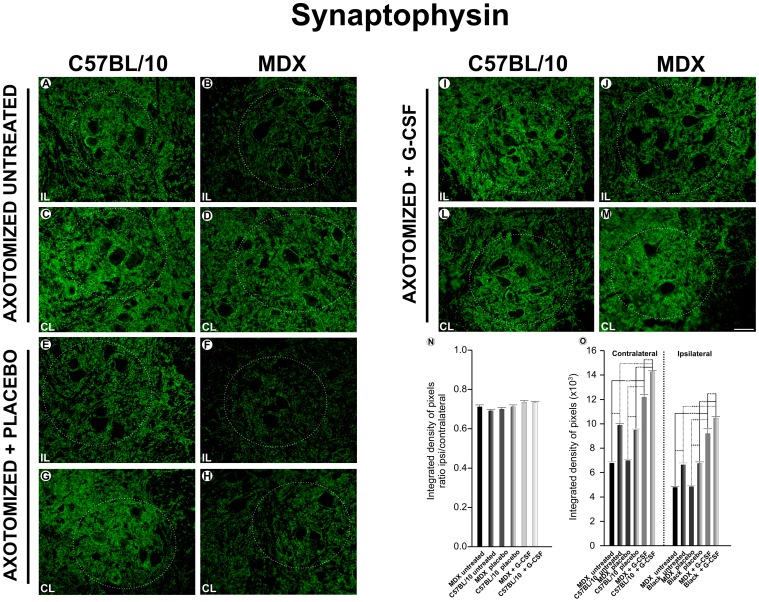
Anti-synaptophysin immunostaining one week after axotomy. **A** and **C**, the ipsilateral (IL) and contralateral ventral column of the spinal cord of the C57BL/10 strain seven days after axotomy; **B** and **D**, the ipsilateral and contralateral ventral column of the spinal cord of the MDX strain seven days after axotomy; **E** and **G**, the ipsilateral and contralateral of the C57BL/10 ipsilateral+ placebo group seven days after the axotomy; **F** and **H**, the ipsilateral and contralateral of the MDX ipsilateral+ placebo group; **I** and **L**, the ipsilateral and contralateral of the C57BL/10 ipsilateral+ G-CSF group; **J** and **M,** the ipsilateral and contralateral of the MDX ipsilateral+ G-CSF group. **N** and **O**, the quantitative analysis of the integrated density of pixels between the ipsi/contralateral ratio and right and left sides, respectively. The circled areas show the motor nucleus of the sciatic nerve and alpha-motoneurons of each lineage. In all experiments: n = 5. In **A–M**, magnification, *X200* (scale bar, 50 µm). The alpha-motoneurons were quantified in 3 distinct fields along the lumbar intumescence. In **N**, ***p<0.001 *vs*. CT, values are means ± SEM.

Figure 2 shows the immunoreactivity for synaptophysin in the ventrolateral motor nucleus of the anterior horn of the spinal cord at seven days after axotomy of the sciatic nerve (for ipsi and contralateral sides). The quantitative data revealed that the G-CSF treatment enhanced the expression of synaptophysin in the nonlesioned spinal cords (Figure S2). Such an increase in immunoreactivity was proportionally greater in the C57BL/6J mice than in the MDX animals.

After treatment with G-CSF, the MDX mice exhibited an approximately 80% increased expression of synaptophysin compared with the untreated group. This same pattern was observed in the C57BL/10 strain and there was a significant increase in the expression of synaptophysin, approximately 53% after treatment. Even accounting for the increased expression of synaptophysin after treatment with G-CSF, the MDX mice showed a significantly lower expression of synaptophysin than the C57BL/10 mice.

In the axotomized groups, the MDX mice showed a significant decrease in synaptophysin expression on the contralateral side to the lesion compared with the C57BL/10 mice. The MDX mice showed decreased immunoreactivity against synaptophysin on the ipsilateral side. Additionally, among the MDX mice, the G-CSF treatment led to a greater preservation of synaptic inputs after injury compared with the untreated and placebo groups. Accordingly, in the C57BL/10 strain, an increase of synaptophysin immunolabeling could be observed after treatment. The analysis of the ipsi/contralateral ratio indicated that there was no difference between the groups and strains studied. All of the quantitative data that were described in this section are presented as supporting information in Table S1.

### Reactive Astrogliosis after Treatment with G-CSF

An increase in GFAP immunoreactivity was observed in the nonlesioned/untreated group of the MDX mice compared with the same group of C57BL/10 mice. After treatment with G-CSF, GFAP immunolabeling increased in both strains, although the MDX mice maintained a greater expression than the C57BL/10 mice (Figure S3).

**Figure 3:**
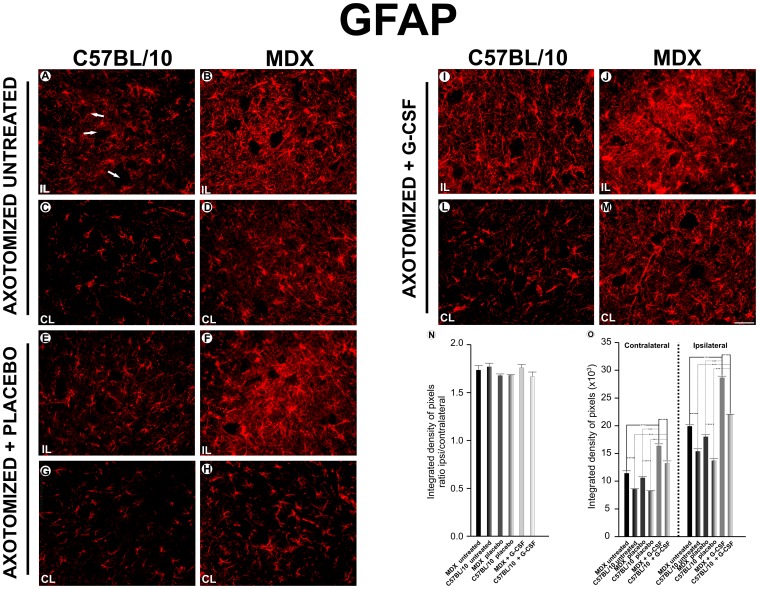
Anti-GFAP immunostaining one week after axotomy. **A** and **C**, the ipsilateral (IL) and contralateral ventral column of the spinal cord of the C57BL/10 strain seven days after axotomy; **B** and **D**, the ipsilateral and contralateral ventral column of the spinal cord of the MDX seven days after axotomy; **E** and **G**, the ipsilateral and contralateral of the C57BL/10 ipsilateral+ placebo group seven days after the axotomy; **F** and **H**, the ipsilateral and contralateral of the MDX ipsilateral+ placebo group; **I** and **L**, the ipsilateral and contralateral of the C57BL/10 ipsilateral+ G-CSF group; **J** and **M**, the ipsilateral and contralateral of the MDX ipsilateral+ G-CSF group. In all experiments: n = 5. In **A–M**, magnification, *X200* (scale bar, 50 µm).The alpha-motoneurons were quantified in 3 distinct fields along the lumbar intumescence. In **N**, ***p<0.001 *vs*. CT, values are means ± SEM.

In both strains, the ipsilateral side of the axotomized/untreated groups presented increased immunoreactivity compared with the contralateral side (Figure 3). Additionally, contralateral to the lesion, the MDX strain showed significantly greater upregulation of labeling than the C57BL/10 strain. The MDX mice showed approximately 31% more upregulation than the C57BL/10 mice. In the placebo group, the MDX mice’s increase was 30% greater than the C57BL/10 mice. The treated MDX group presented an increase of approximately 24% compared with the C57BL/10 strain.

The ipsilateral side of the axotomized groups showed a significant increase in GFAP expression after the lesion, primarily among the MDX strain. In the untreated group, there was an increase of approximately 30% compared with the C57BL/10 group. The placebo group showed an increase of approximately 32%, and the G-CSF treated group displayed an increase of 31%. A comparison of the G-CSF treated group with the untreated and placebo groups found an increased expression of GFAP in both strains among the treated group. The C57BL/10 mice that were treated with G-CSF displayed an increase of approximately 43% compared with the untreated group and 60% compared with the placebo group.

An analysis of the ipsi/contralateral ratio among the different experimental groups indicated no difference between the groups and strains studied. All of the quantitative data that were described in this section are presented as supporting information (Table S2).

### Immunoreactivity for IBA-1 after Treatment with G-CSF

The immunoreactivity against IBA-1 remained at low levels in the nonlesioned/untreated and nonlesioned+G-CSF groups. (Figure S4).

**Figure 4:**
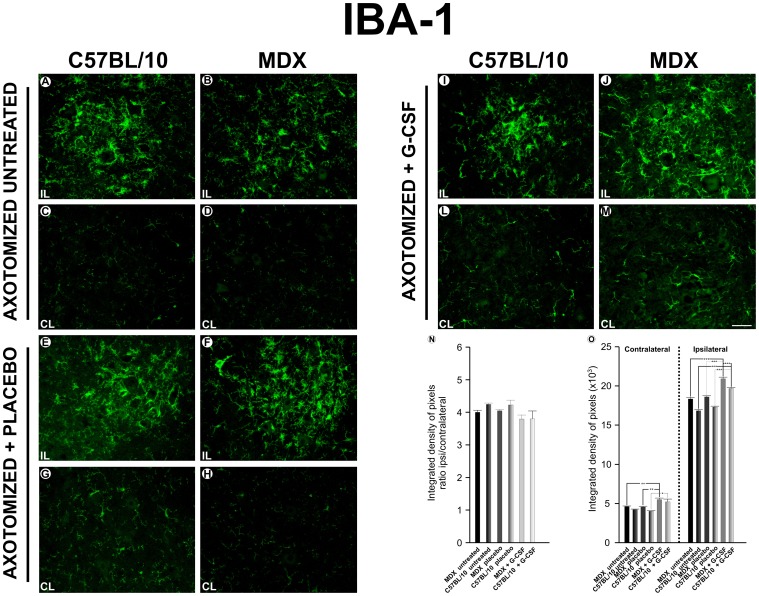
Anti-IBA1 immunostaining one week after axotomy. **A** and **C**, the ipsilateral (IL) and contralateral ventral column of the spinal cord of the C57BL/10 strain seven days after axotomy; **B** and **D**, the ipsilateral and contralateral ventral column of the spinal cord of the MDX seven days after axotomy; **E** and **G**, the ipsilateral and contralateral of the C57BL/10ipsilateral+ placebo group seven days after the axotomy; **F** and **H**, the ipsilateral and contralateral of the MDX ipsilateral+ placebo group; **I** and **L**, the ipsilateral and contralateral of the C57BL/10 ipsilateral+ G-CSF group; **J** and **M**, the ipsilateral and contra lateral of the MDX ipsilateral+ G-CSF group. In all experiments: n = 5. In **A–M**, magnification, *X200* (scale bar, 50 µm).The alpha-motoneurons were quantified in 3 distinct fields along the lumbar intumescence. In **N**, *p<0.05, **p<0.01, ***p<0.001 *vs*. CT, values are means ± SEM.

After axotomy, the immunoreactivity displayed by the contralateral/untreated and contralateral+placebo groups was similar to the immunoreactivity displayed by the non lesioned/untreated group (Figure 4). In the contralateral+G-CSF group, no significant differences were found between the strains. However, for the contralateral side, the MDX mice that were treated with G-CSF presented a significant increase in immunoreactivity compared with the other two groups (21% greater than the untreated group and 20% greater than the placebo group). A non-significant increase in the expression of IBA-I was depicted after axotomy. Interestingly, for the ipsilateral side, the G-CSF-treated animals from both strains displayed an increased expression of IBA-I (15% greater than the untreated group and 12% greater than the placebo group in the MDX mice and 17% greater than the untreated group and 14% greater than the placebo group for the C57BL/10 mice). All of the quantitative data are presented as supporting information (Table S2).

### Ultrastructural Changes in Spinal Cord Injury after Sciatic Nerve Axotomy and Subsequent Treatment with G-CSF

**Figure 5:**
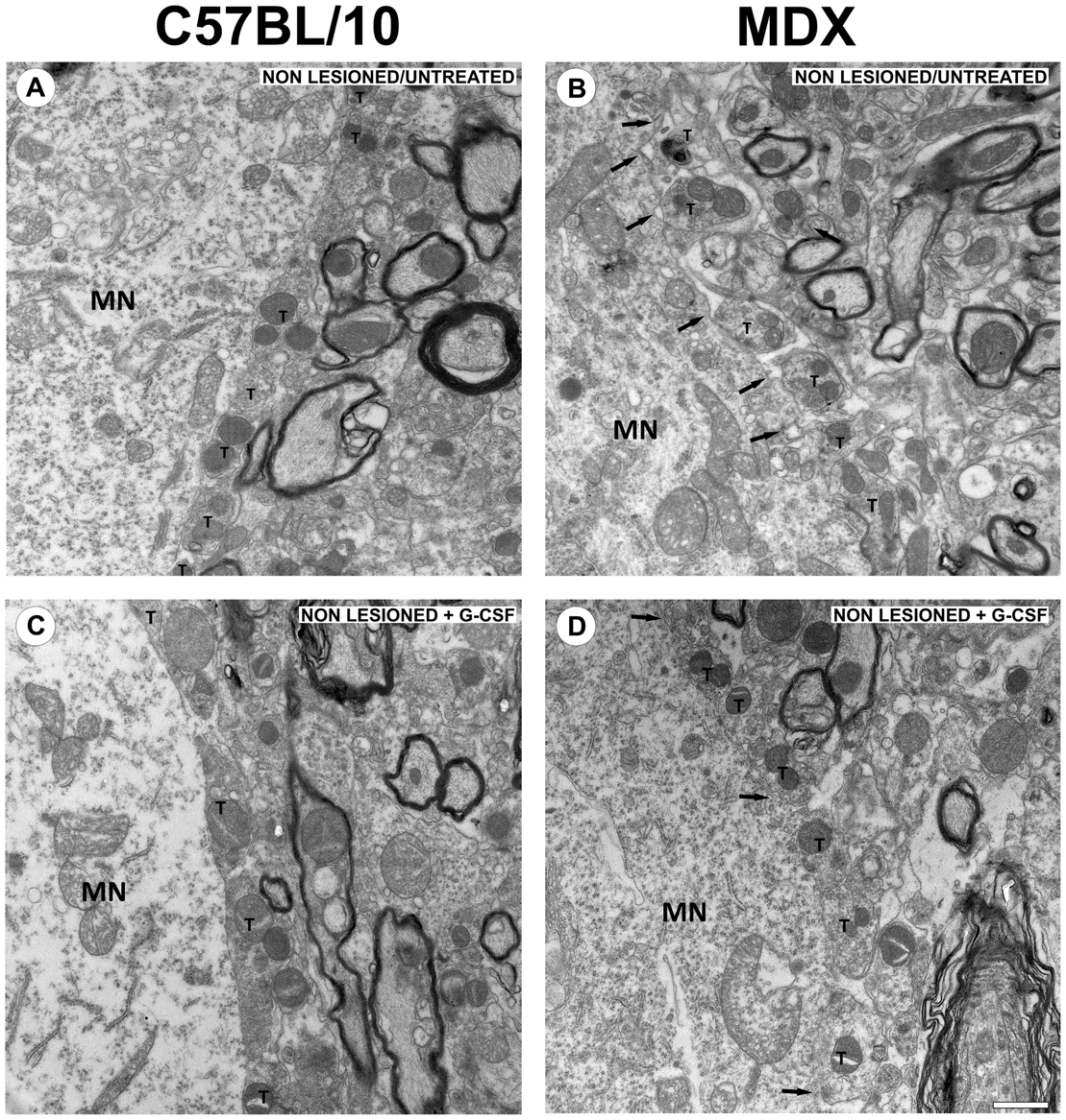
Synaptic covering of non lesioned mice. (**A–D**) Transmission electron microscopy images. (**A**) synaptic covering of non lesioned/untreated neuron of the C57BL/10 strain. (**B**) synaptic covering of non lesioned/untreated neuron of the MDX strain. (**C**) synaptic covering of non lesioned +G-CSF neuron of the C57BL/10 strain. (**D**) synaptic covering of non lesioned +G-CSF neuron of the MDX strain. Note some retractions and astrocytic projections (arrows) between the terminals and the membrane of the neuron in Figure **B**. In all experiments: n = 5. In **A–D**, magnification, *X4800* (scale bar, 1 µm). In **B** and **D,** the arrows indicate that the alpha-motoneurons are partially detached, with a reduced area of apposition. In **A–D**, **T** indicates the presynaptic terminals and **MN** indicates the alpha-motoneuron’s cell body.

**Figure 6:**
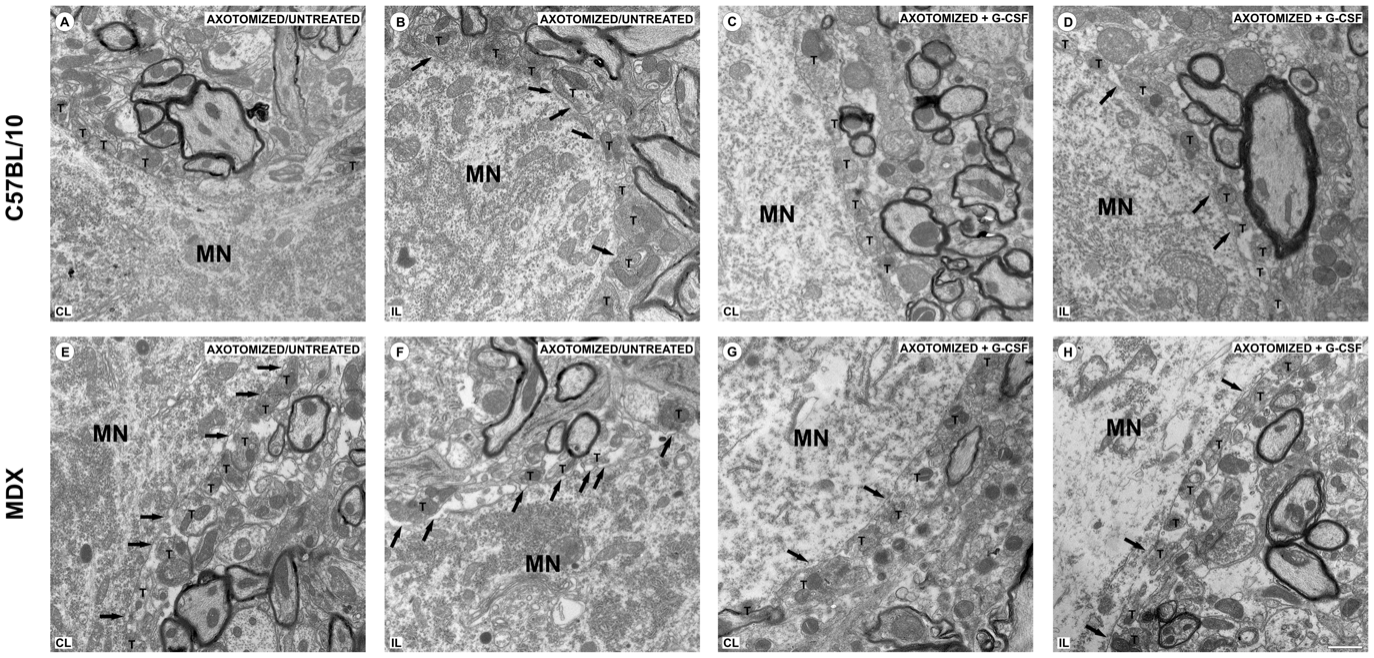
Surface ultrastructure of alpha-motoneurons one week after axotomy. (**A–H**) Transmission electron microscopy images. (**A**) synaptic covering of neuron of the C57BL/10 contralateral/untreated group. (**B**) synaptic covering of neuron of the C57BL/10 ipsilateral/untreated group. The astrocytic retractions and projections in this group express a significant increase compared with the contralateral side of the same strain one week after injury. (**C**) synaptic covering of neuron of the C57BL/10 contralateral+G-CSF group. (**D**) synaptic covering of neuron of the C57BL/10 ipsilateral+G-CSF group. (**E**) synaptic covering of neuron of the MDX contralateral/untreated group. (**F**) synaptic covering of neuron of the MDX ipsilateral/untreated group. (**G**) synaptic covering of neuron of the MDX contralateral+ G-CSF group. (**H**) synaptic covering of neuron of the MDX ipsilateral+ G-CSF group. Note the retraction and gliosis processes exacerbated in the untreated groups. In the groups treated with G-CSF, the retraction processes must be smaller. In all experiments: n = 5. In **A–H**, magnification, *X4800* (scale bar, 1 µm). In **B–H,** the arrows indicate that the alpha-motoneurons are partially detached, with a reduced area of apposition. In **A–H**, **T** indicates the presynaptic terminals and **MN** indicates alpha-motoneurons cell body.

All of the motoneurons considered for this analysis displayed at least one cholinergic pre-synaptic terminal (type C) in apposition to the neuronal membrane surface. The ultrastructural analysis of the samples from the non lesioned animals revealed a broad and even covering on the membrane surface (Figure 5). However, in MDX mice, some ultrastructural abnormalities were observed concerning the pre-synaptic terminals located in opposition to the spinal alpha-motoneurons. Such ultrastructural changes were found less frequently in the non lesioned MDX mice that were treated with G-CSF. Moreover, the neurons that were affected by the lesion showed changes that indicated chromatolysis, such as a displacement of the nucleus to the periphery of the cell body and a decrease in cytoplasm electrondensity that was consistent with the dissolution of Nissl corpuscles. Thin glial projections that were in contact with the postsynaptic membrane were also frequently identified, filling the space between the synaptic terminal and postsynaptic membrane (Figure 6). These structures were identified as astrocytes because of their low electrodensity.

**Figure 7:**
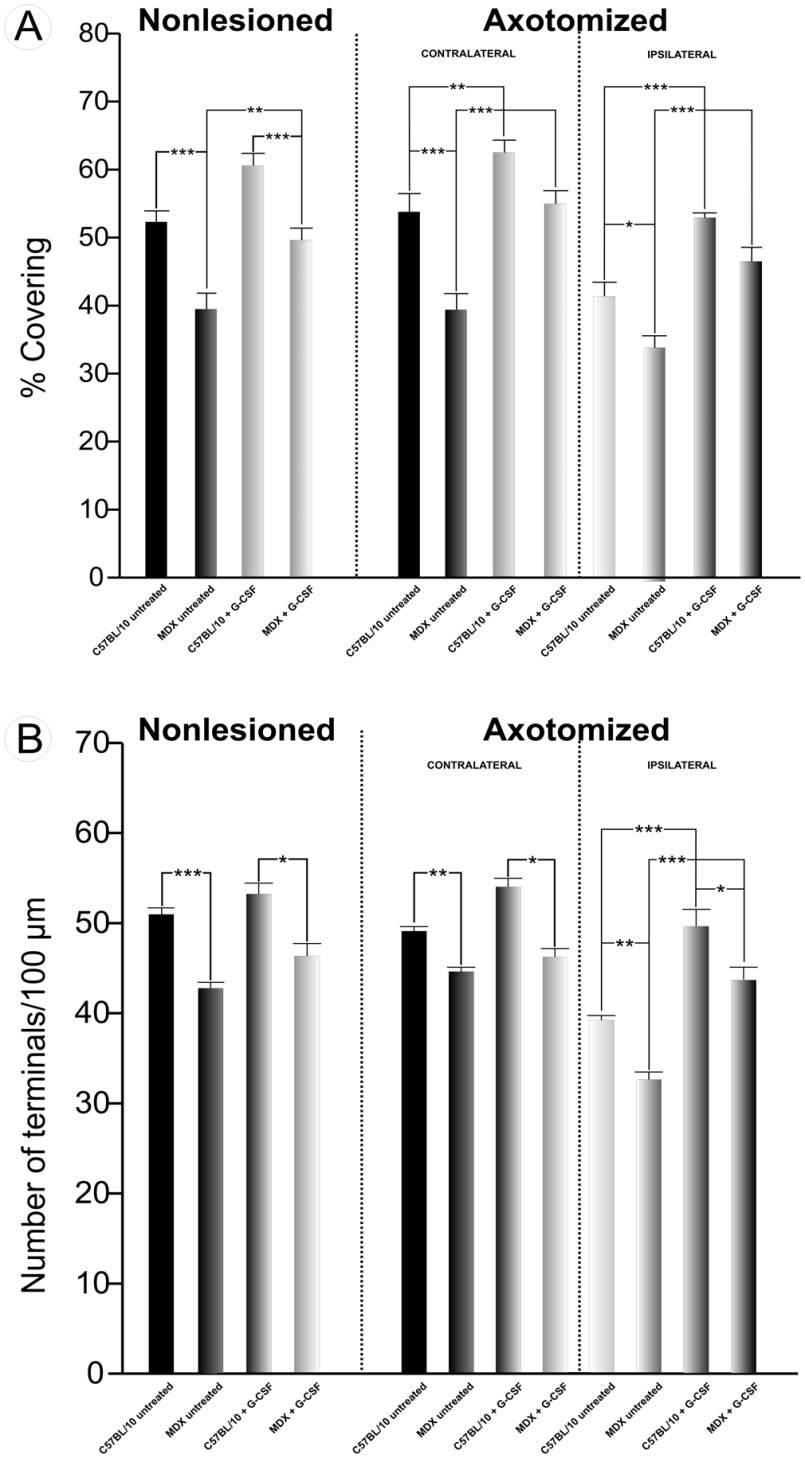
Quantitative analysis. (**A**) quantitative analysis of the ultrastructure of the synapses showing the percentage of synaptic covering in the non lesioned, ipsilateral and contralateral, untreated and treated with G-CSF groups. (**B**) quantitative analysis of the ultrastructure of the number of presynaptic terminals in the neuronal membrane apposition/100 µm between the non lesioned, ipsilateral and contralateral, untreated and treated with G-CSF groups. In all experiments: n = 5. In **A** and **B**, *p<0.05, **p<0.01, ***p<0.001 *vs*. CT, values are means ± SEM.

All of the ultrastructural changes described after axotomy were exacerbated in the MDX mice. A quantitative analysis of the untreated MDX mice showed a reduction in synaptic covering compared with the C57BL/10 mice (Figure 7A). The differences were significant for the ipsilateral/untreated group, resulting in a 21% reduction in synaptic covering in the MDX strain. Among the nonlesioned/untreated and contralateral/untreated specimens, those samples from the MDX mice showed significantly less synaptic covering than the C57BL/10 strain. Additionally, no significant differences were found between groups in the analysis of the nonlesioned/untreated MDX and axotomized contralateral/untreated MDX groups. However, when these two groups were compared with the axotomized ipsilateral/untreated MDX group, a significant reduction in synaptic covering could be observed along the injured side. Interestingly, an analysis of the G-CSF-treated mice showed important preservation of the synaptic covering at one week after sciatic nerve transection (Table S3).

It is important to emphasize that a significant increase in synaptic covering was also observed in the non lesioned MDX mice that were treated with G-CSF. The same increase in synaptic covering was observed for the C57BL/10 strain.

**Figure 8:**
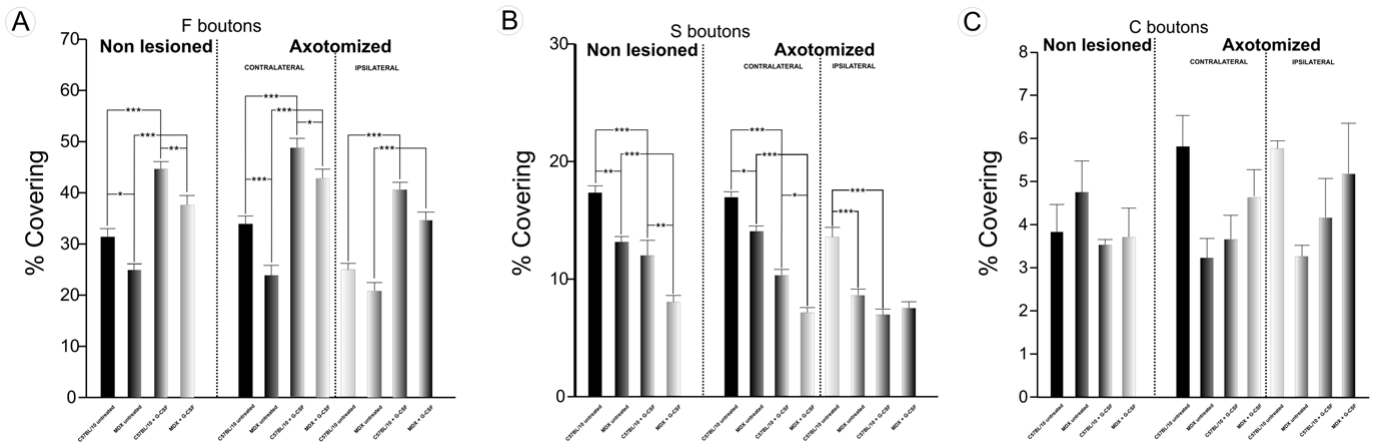
Quantitative analysis. Representation of ultrastructural quantitative analysis of the synaptic covering percentage. (**A**) quantitative analysis of the synaptic covering percentage of F terminals. (**B**) quantitative analysis of the synaptic covering percentage of S terminals. (**C)** quantitative analysis of the synaptic covering percentage of C terminals. In all experiments: n = 5. In **A** and **B**, *p<0.05, **p<0.01, ***p<0.001 *vs*. CT, values are means ± SEM.

The quantitative analysis of the F bouton covering (boutons containing flattened vesicles with glycine as the neurotransmitter) revealed a significantly smaller number in both strains after transection of the sciatic nerve. This decrease was more pronounced in the MDX strain than in the C57BL/10 strain (Figure 8A). However, the MDX mice that were treated with G-CSF had a greater number of F presynaptic terminals after axotomy than the untreated mice. An increased covering of F terminals after treatment with G-CSF was also observed in the C57BL/10 strain.

The percentage of S boutons (boutons containing spherical vesicles with glutamate as the neurotransmitter) showed significant differences between the groups. The percentage was lower in the MDX mice than in the C57BL/10 mice (Figure 8B). The analysis of the nonlesioned/untreated and the contralateral/untreated groups showed no significant differences between groups for the MDX strain. This lack of difference was also observed in the same groups of the C57/BL10 strain. However, treatment with G-CSF promoted a significant reduction in the percentage of S boutons in apposition to the neuronal membrane for both strains. All of the numerical data concerning synaptic covering are presented in the supporting information (Table S4).

The number of boutons/100 µm in apposition to the neuronal membrane in the MDX ipsilateral/untreated group was significantly lower than the C57BL/10 (Figure 7B). However, mice that were treated with G-CSF lost fewer synaptic contacts one week after injury. This preservation of presynaptic boutons in the mice that were treated with G-CSF was more evident when compared with untreated mice. In the non lesioned/untreated groups, the axotomized contralateral/untreated side and axotomized contralateral+G-CSF side, a significant reduction in the number of boutons in apposition to the neuronal membrane was observed in the MDX strain compared with the C57BL/10 strain (Figure7B).

An analysis of the number of F boutons/100 µm of neuronal membrane in both strains showed a significant difference between the nonlesioned/untreated and contralateral/untreated groups, with a lower number in the MDX mice than in the C57BL/10 mice (Figure S5 - A). However, no significant differences were observed between the MDX ipsilateral/untreated and the C57/BL10 ipsilateral/untreated groups. After treatment with G-CSF, a smaller decrease in F boutons after axotomy was observed. Consequently, a significant increase in the number of F terminals in apposition to the neuronal membrane/100 µm was observed for the G-CSF groups compared with the untreated groups (Figure S5–A).

An analysis of the number of S boutons in apposition/100 µm of neuronal membrane showed a significant reduction between the strains after lesion (Figure S5 - B). For the MDX mice, there was no significant difference between the nonlesioned/untreated mice and the contralateral/untreated mice. However, a comparison between the non lesioned/untreated, axotomized contralateral/untreated side and the axotomized ipsilateral/untreated side showed a significant reduction of S boutons in apposition to the neuronal membrane/100 µm. After treatment with G-CSF, a lower number of S boutons in apposition to the neuronal membrane/100 µm were observed in the G-CSF group compared with the untreated groups in both strains (Figure S5–B). All of the numerical data concerning the number of boutons/100 µm are presented in the supporting information (Table S5).

The gaps between the clusters of terminals that were located in apposition to the postsynaptic membrane were measured to calculate the frequency of distribution at intervals of 1 µm. This analysis indicated a higher frequency of larger spaces between synaptic terminals in the MDX nonlesioned/untreated and MDX contralateral/untreated groups compared with the C57BL/10 mice. This difference was most evident after axotomy in the untreated MDX mice due to the increased synaptic retraction that occurred. Even with the exacerbated retraction of synaptic boutons, the terminals still remained in small groups, which indicated selectivity in the retraction process. Among the groups that were treated with G-CSF, an increase in the number of spaces between the presynaptic terminals of smaller size was noticed, and a reduction was observed in the number of spaces between the presynaptic terminals of larger size. This pattern was observed in both strains studied, although it was more evident in the MDX animals than in the C57BL/10 animals (Figure S6).

## Discussion

Muscular dystrophy is a multifaceted disease that develops in childhood and evolves into paralysis and death. Most of the knowledge about MD focused on the muscular system because this system is the primary target for potential treatments. Few studies have been devoted to a better understanding of the absence of dystrophy in the central nervous system (CNS). The current authors have previously shown that MDX mice displayed significant changes in the spinal cord circuits during the course of Duchenne muscular dystrophy and that spinal motoneurons were affected [15]. By studying the ultrastructure of the inputs to the alpha-motoneurons, it was possible to show a retrograde loss of inputs similar to the pattern triggered by peripheral nerve lesion. The hypothesis was raised that adjustments occur in the synaptic spinal motoneurons during the process of muscle degeneration/regeneration, thereby promoting a partial disconnection of the peripheral nerve with its target.

The presentation of a lower expression of synaptophysin among the MDX mice indicates that this reduction of synapses in the alpha-motoneurons could be due to a partial disconnection between the motoneuron and the target organ during the cycles of degeneration/regeneration that occur from the second postnatal week. Moreover, in MDX mice, there was a decrease in the number of cells in the corticospinal tract [12]. This decrease in cells could also decrease the number of inputs to the alpha-motoneurons and thus cause a reduction in synaptic covering to the cell bodies. The same authors have also shown changes in the architecture of the corticospinal tract in MDX mice at 3 months of age [12]. These changes were related to a reduction in the size and number of axons. Nevertheless, the number of motoneurons that were retrogradely labeled in the lumbar ventral horn after injections of wheat germ agglutinin in horseradish peroxidase (WGA-HRP) was very similar in the MDX mice and the C57BL/10 mice. Bearing in mind the possible repercussions of the muscle degeneration/regeneration that occurred in the spinal cord microenvironment, the pre-treatment and post-lesion administration of G-CSF has been addressed in this study as a pharmacological strategy to retard the evolution of the disease in the CNS and to improve the regenerative potential of the lesioned neurons after axotomy.

The overall distribution of the spinal cord synapses was studied in terms of the expression of synaptophysin. There was reduced immunoreactivity in the MDX strain. The MDX mice showed a significant decrease in the expression of synaptophysin after the lesion, approximately 25% lower compared with the C57BL/10 strain, which indicated a decreased ability to respond to injury. However, the treated group displayed an increased expression of synaptophysin compared with the placebo and untreated groups, and the pattern was more pronounced in the MDX mice than the C57BL/10 mice (an increase of approximately 80% compared with the untreated group and 79% compared with the placebo group). This difference may indicate that the G-CSF treatment is neuroprotective by increasing the synaptic stability in the spinal cord and partially counteracting the deleterious effects of the disease. The effectiveness of G-CSF may also be related to the expression of its receptor by spinal neurons, which are the primary targets of such cytokines in the CNS [50].

As demonstrated after nerve injury, MHC I plays a key role in the selective stabilization of inhibitory synapses and contributes to the specificity of the input rearrangement [15], [51]–[53]. SABHA et al. [54] correlated the increased expression of MHC I with the intensification of the synaptic elimination process after seven days of peripheral axotomy in the spinal cord microenvironment. This correlation appeared to be valid in the present work, although the MDX mice presented a lower expression of MHC I than the C57BL/10 mice. Thus, stimulating increased levels of the MHC I molecule in MDX mice via treatment with G-CSF may be a strategy to improve the animals’ response to injury and to increase the stability of the synapses in the spinal cord during the course of the disease. The results herein indicate a way to improve the response to injury in MDX mice, reinforcing the idea that pharmacological treatments may delay worsening of the disease and preserve the motor network.

The ultrastructural analysis was consistent with the results discussed above in that the alpha-motoneurons showed synaptic loss in the untreated MDX. The G-CSF treatment reduced such a loss and also decreased the elimination of terminals after axotomy. A quantitative analysis of the synaptic covering for each type of presynaptic terminal revealed that there was a significant decrease in inhibitory (F type) and excitatory (S type) terminals in the MDX untreated groups (non lesioned, contralateral and ipsilateral). Keeping in mind the differences between the groups, a difference was identified in the covering for the F and S terminals before and after axotomy, such that the S type was the most affected type. In mice that were treated with G-CSF, an increased percentage of F-type terminal covering was observed (approximately 70% for the ipsilateral group). A significant reduction in the percentage covering of S-type terminals was observed after the peripheral axotomy (approximately 11% for a). Such results indicate that treatment with G-CSF may be able to promote a selective synaptic elimination process with a preference for the maintenance of F-type synaptic contacts. This preference may lead to neuroprotection because excitotoxicity is avoided due to most of the glutamatergic terminals being retracted from the motoneuron cell bodies. This result is in line with previous data [51], [55].

In addition to the prevention of excitotoxicity, the amount of presynaptic terminals/100 µm in apposition to the neuronal membrane was also increased in the MDX mice after G-CSF treatment. Therefore, we suggest that the pretreatment with G-CSF provided neuroprotection to the spinal cord microenvironment during the course of Duchenne muscular dystrophy. This protective effect was reflected in the preservation of spinal cord motoneuron circuits and in the reduction of the number of partially retracted synapses.

The expression of GFAP and IBA-1 demonstrated that glial reactivity was further enhanced in MDX mice than in the control animals. Surprisingly, the treatment with G-CSF increased the immunolabeling of positive astrocytes for GFAP and IBA-1 for microglial cells, which possibly indicated a higher astrogliosis and immunity response showed by the activated microglia. Although astrocytes and microglia may play a negative role on synaptic plasticity and neuronal regeneration [56], [57], new evidences opposing such ideas have been demonstrated. Recent studies have shown that astrocytes may develop a positive role during nervous system regeneration [58]. Among such effects, neuroprotection [59], reestablishment of the blood-brain barrier [60], [61], uptake of K+ [62], [63], and regulation of glutamate levels [64] may be highlighted. Additionally, the production and release of important antioxidant factors [65]–[70] and the secretion of neurotrophic factors [71] are also of great importance. Therefore, astroglial reactivity, which was stimulated by the G-CSF treatment, may play a key role in the reorganization of the neuronal microenvironment surrounding the motoneurons, especially in MDX mice. Similarly, recent studies showed that microglia also synthesize and release neurotrophic factors after injury to the CNS by developing a role in repair and contributing to the regenerative process in the CNS [72]–[74]. CULHEIM and THAMS [75] suggested that microglia and MHC I expression play an important role in the removal synaptic terminals after nerve axotomy. Our results indicate that treatment with G-CSF stimulates the glial reaction without an exacerbation of synaptic loss. This pattern may be interpreted as the reactive glia being driven towards a neuroprotective role by contributing to the stability of the surrounding microenvironment of the motoneurons.

Taken together, the present data indicate that the G-CSF treatment is neuroprotective, acting at the spinal cord level and increasing the stability of F-type terminals. The MDX mice displayed a close-to-normal synaptic plasticity in response to injury after the G-CSF administration. Overall, our data suggest that such an immunomodulatory drug may contribute to delaying the course of DMD by preserving the spinal cord microenvironment.

## Supporting Information

Figure S1 **Anti-MHC I immunostaining on non lesioned mice.** **A**, the ventral column of the spinal cord of the C57BL/10 strain without treatment; **B**, ventral column of the spinal cord of the MDX strain without treatment; **C**, the ventral column of the spinal cord of the C57BL/10 strain treated with G-CSF; **D**, ventral column of the spinal cord of the MDX strain treated with G-CSF. In all experiments: n = 5. In **A–D**, magnification, *X200* (scale bar, 50 µm).
(TIF)

Figure S2 **Anti-synaptophysin immunostaining on non lesioned mice. A,** the ventral column of the spinal cord of the C57BL/10 strain without treatment; **B**, the ventral column of the spinal cord of the MDX strain without treatment; **C**, the ventral column of the spinal cord of theC57BL/10 strain treated with G-CSF; **D**, the ventral column of the spinal cord of the MDX strain treated with G-CSF. **E**, quantitative analysis of the integrated density of pixels between the right and left sides. The circled areas show the motor nucleus of the sciatic nerve and alpha-motoneurons of each lineage. In all experiments: n = 5. In **A–M**, magnification, *X200* (scale bar, 50 µm). The alpha-motoneurons were quantified in 3 distinct fields along the lumbar intumescence. In **E**, **p<0.01 *vs*. CT, values are means ± SEM.
(TIF)

Figure S3 **Anti-GFAP immunostaining on non lesioned mice. A,** the ventral column of the spinal cord of the C57BL/10 strain without treatment; **B**, ventral column of the spinal cord of the MDX strain without treatment; **C**, the ventral column of the spinal cord of the C57BL/10 strain treated with G-CSF; **D**, ventral column of the spinal cord of the MDX strain treated with G-CSF. **E**, quantitative analysis of the integrated density of pixels between the right and left sides. In all experiments: n = 5. In **A–D**, magnification, *X200* (scale bar, 50 µm). The alpha-motoneurons were quantified in 3 distinct fields along the lumbar intumescence. In **E**, ***p<0.001 *vs*. CT, values are means ± SEM.
(TIF)

Figure S4 **Anti-IBA1 immunostaining on non lesioned mice. A,** the ventral column of the spinal cord of the C57BL/10 strain without treatment; **B**, ventral column of the spinal cord of the MDX strain without treatment; **C**, the ventral column of the spinal cord of the C57BL/10 strain treated with G-CSF; **D**, the ventral column of the spinal cord of the MDX strain treated with G-CSF. **E**, quantitative analysis of the integrated density of pixels between the right and left sides. In all experiments: n = 5. In **A–D**, magnification, *X200* (scale bar, 50 µm). The alpha-motoneurons were quantified in 3 distinct fields along the lumbar intumescence. In **E**, p>0.05 *vs*. CT, values are means ± SEM.
(TIF)

Figure S5 **Synapse quantitative analysis.** Representation of the ultrastructural quantitative analysis of the number of presynaptic terminals in neuronal membrane apposition/100 µm. (**A**) quantitative analysis of the number of F terminals in neuronal membrane apposition/100 µm. (**B**) quantitative analysis of the number of S terminals in neuronal membrane apposition/100 µm. (**C)** quantitative analysis of the number of C terminals in neuronal membrane apposition/100 µm. In all experiments: n = 5. In **A**, *p<0,05, **p<0,01, ***p<0.001 *vs*. CT. In **B,** **p<0.01, ***p<0.001 *vs*. CT, values are means ± SEM.
(TIF)

Figure S6 **Frequency distribution (in microns) of the spaces between the synaptic terminals retracted along the membrane surface of the motoneurons.** (**A**) C57Bl/10 non lesioned/untreated. (**B**) MDX non lesioned/untreated. (**C**) C57Bl/10 non lesioned+G-CSF. (**D**) MDX non lesioned+G-CSF. (**E**) C57Bl/10 contralateral/non lesioned. (**F**) MDX contralateral/non lesioned. (**G**) C57Bl/10 contralateral+G-CSF. (**H**)MDX contralateral+G-CSF. (**I**) C57Bl/10 ipsilateral/non lesioned. (**J**) MDX ipsilateral/non lesioned. (**L**) C57Bl/10 ipsilateral+G-CSF. (**M**) MDX ipsilateral+G-CSF. In all experiments: n = 5. In **A–M**, values are means ± SEM.
(TIF)

Table S1 **MHC I and synaptophysin immunolabeling quantification in non lesioned, untreated, placebo and treated with G-CSF groups.** The data represent the mean value of the integrated density of pixels measured ± SEM. The different letters in each column represent the significant differences among the experimental groups.
(DOCX)

Table S2 **GFAP I and IBA 1 immunolabeling quantification in non lesioned, untreated, placebo and treated with G-CSF groups.** The data represent the mean value of the integrated density of pixels measured ± SEM. The different letters in each column represent the significant differences among the experimental groups.
(DOCX)

Table S3 **Transmission electron microscopy quantification for the percentage of covering and number of boutons/100 µm in non lesioned, untreated, placebo and treated with G-CSF groups.** The data represent the mean value of the percentage covering and the number of boutons/100 µm ± SEM. The different letters in each column represent the significant differences among the experimental groups.
(DOCX)

Table S4 **Transmission electron microscopy quantification for the percentage of F, S and C boutons’ covering in non lesioned, untreated, placebo and treated with G-CSF groups.** The data represent the mean value of the percentage covering ± SEM. The different letters in each column represent the significant differences among the experimental groups.
(DOCX)

Table S5 **Transmission electron microscopy quantification for the number of boutons/100 µm of F, S and C boutons’ covering in non lesioned, untreated, placebo and treated with G-CSF groups.** The data represent the mean value of the number of boutons/100 µm± SEM. The different letters in each column represent the significant differences among the experimental groups.
(DOCX)
