# Supplementary figures and images for: Granulocyte-Colony Stimulating Factor Improves MDX Mouse Response to Peripheral Nerve Injury
Source: PLoS One. 2012 Aug 13;7(8):e42803. doi: 10.1371/journal.pone.0042803 (PMC3418329; doi:10.1371/journal.pone.0042803)

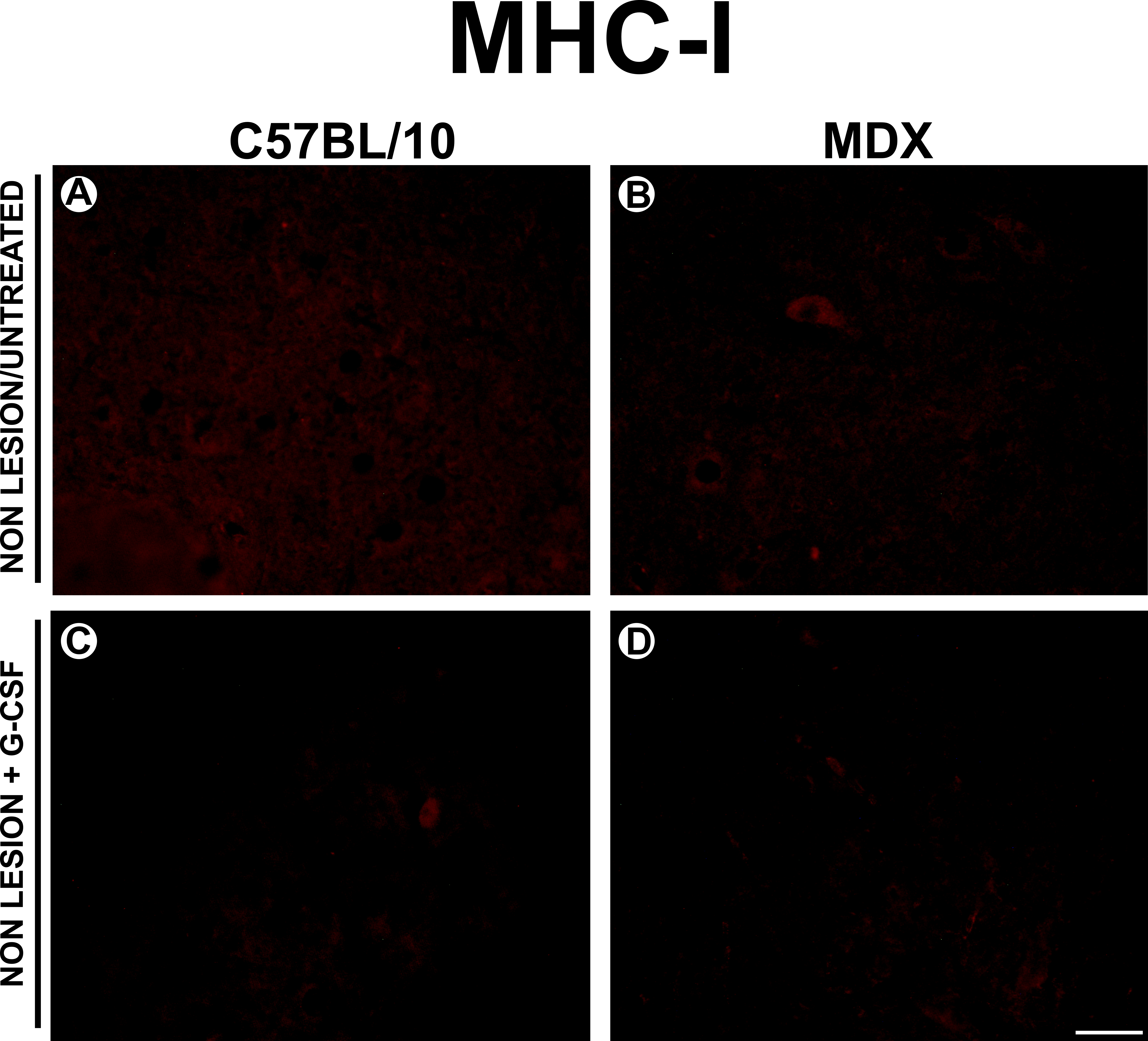

Supplement: Figure S1 — Anti-MHC I immunostaining on non lesioned mice. A, the ventral column of the spinal cord of the C57BL/10 strain without treatment; B, ventral column of the spinal cord of the MDX strain without treatment; C, the ventral column of the spinal cord of the C57BL/10 strain treated with G-CSF; D, ventral column of the spinal cord of the MDX strain treated with G-CSF. In all experiments: n = 5. In A–D, magnification, X200 (scale bar, 50 µm). (TIF) [file pone.0042803.s001.tif]

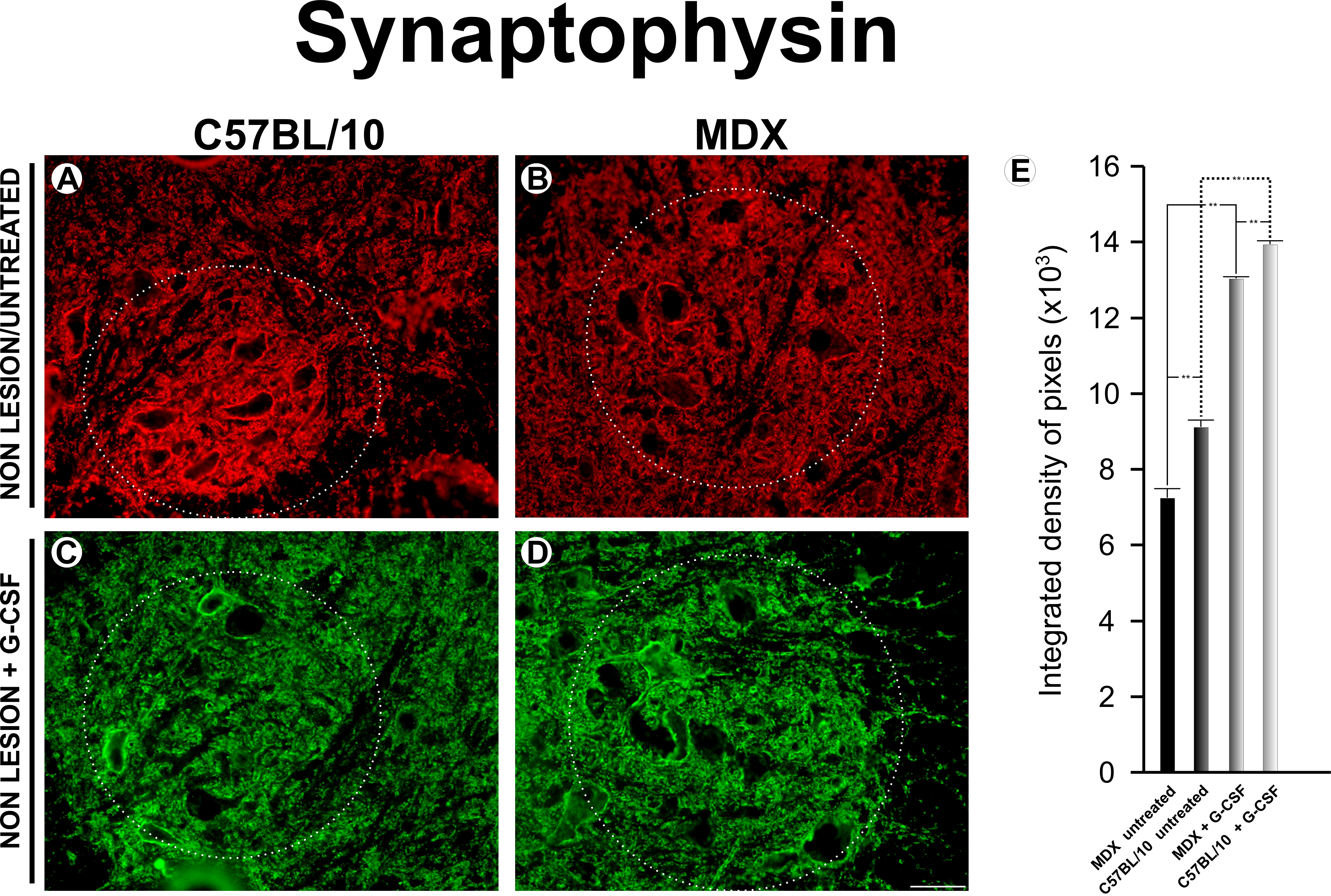

Supplement: Figure S2 — Anti-synaptophysin immunostaining on non lesioned mice. A, the ventral column of the spinal cord of the C57BL/10 strain without treatment; B, the ventral column of the spinal cord of the MDX strain without treatment; C, the ventral column of the spinal cord of theC57BL/10 strain treated with G-CSF; D, the ventral column of the spinal cord of the MDX strain treated with G-CSF. E, quantitative analysis of the integrated density of pixels between the right and left sides. The circled areas show the motor nucleus of the sciatic nerve and alpha-motoneurons of each lineage. In all experiments: n = 5. In A–M, magnification, X200 (scale bar, 50 µm). The alpha-motoneurons were quantified in 3 distinct fields along the lumbar intumescence. In E, **p<0.01 vs. CT, values are means ± SEM. (TIF) [file pone.0042803.s002.tif]

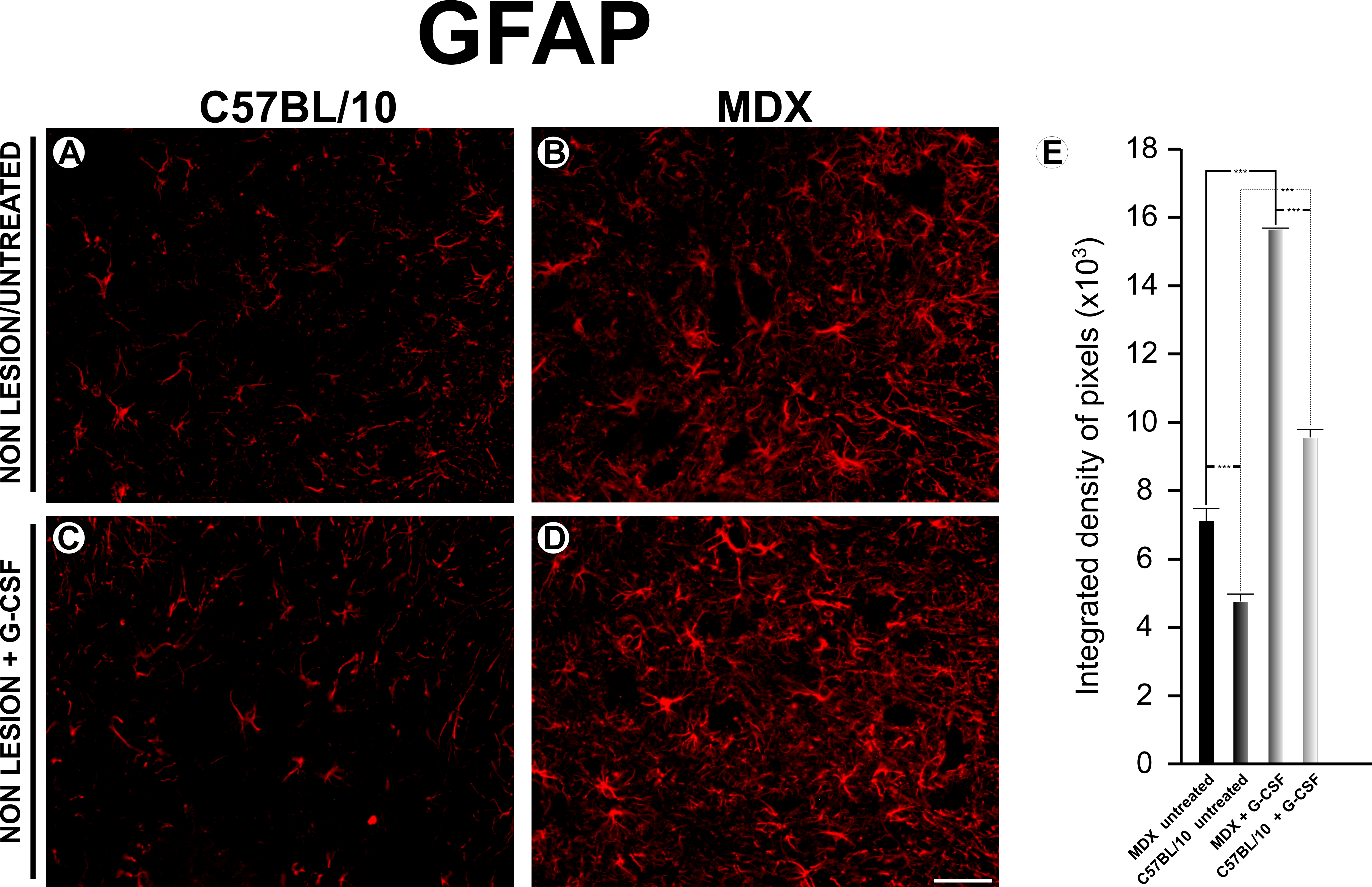

Supplement: Figure S3 — Anti-GFAP immunostaining on non lesioned mice. A, the ventral column of the spinal cord of the C57BL/10 strain without treatment; B, ventral column of the spinal cord of the MDX strain without treatment; C, the ventral column of the spinal cord of the C57BL/10 strain treated with G-CSF; D, ventral column of the spinal cord of the MDX strain treated with G-CSF. E, quantitative analysis of the integrated density of pixels between the right and left sides. In all experiments: n = 5. In A–D, magnification, X200 (scale bar, 50 µm). The alpha-motoneurons were quantified in 3 distinct fields along the lumbar intumescence. In E, ***p<0.001 vs. CT, values are means ± SEM. (TIF) [file pone.0042803.s003.tif]

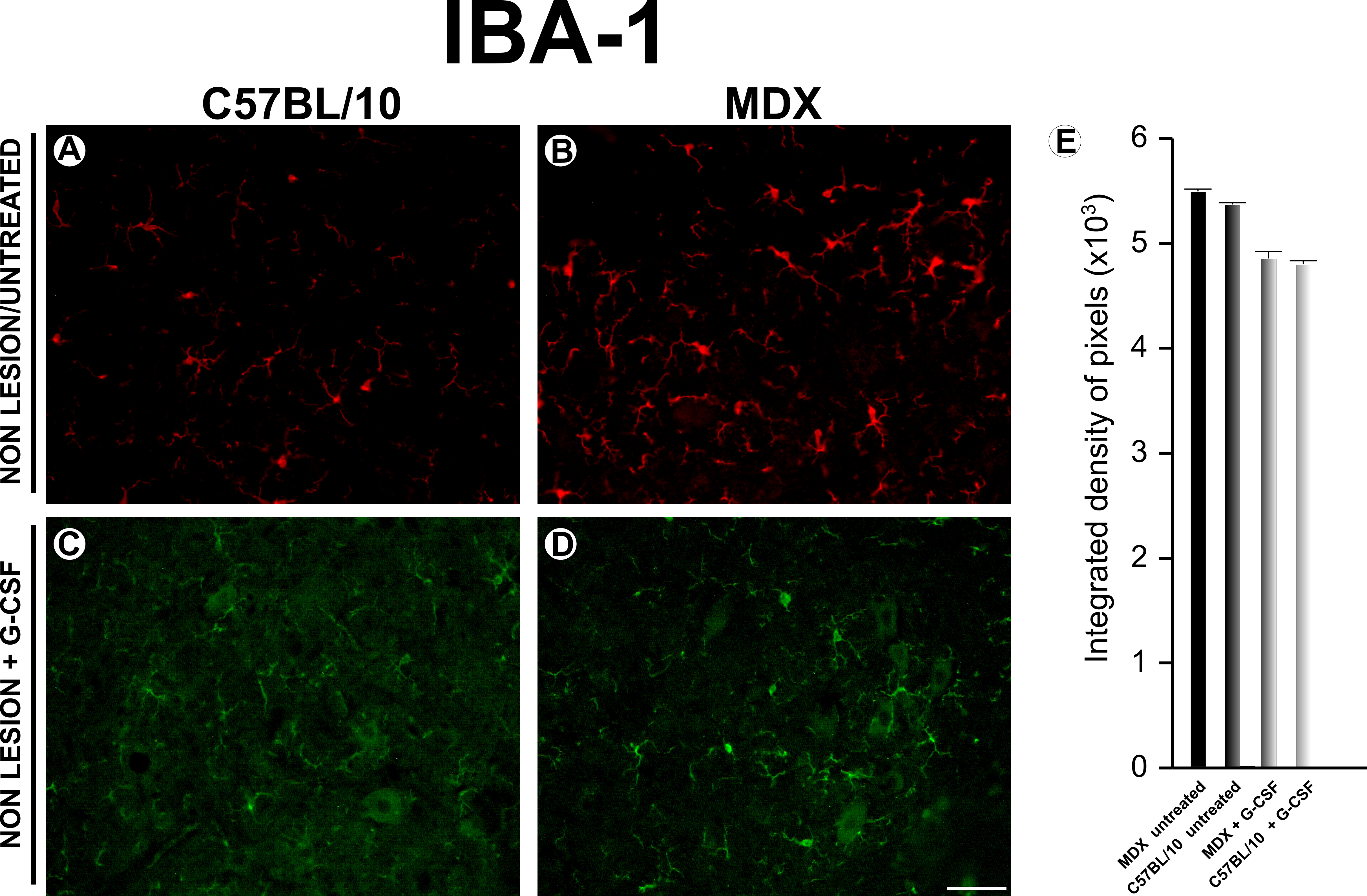

Supplement: Figure S4 — Anti-IBA1 immunostaining on non lesioned mice. A, the ventral column of the spinal cord of the C57BL/10 strain without treatment; B, ventral column of the spinal cord of the MDX strain without treatment; C, the ventral column of the spinal cord of the C57BL/10 strain treated with G-CSF; D, the ventral column of the spinal cord of the MDX strain treated with G-CSF. E, quantitative analysis of the integrated density of pixels between the right and left sides. In all experiments: n = 5. In A–D, magnification, X200 (scale bar, 50 µm). The alpha-motoneurons were quantified in 3 distinct fields along the lumbar intumescence. In E, p>0.05 vs. CT, values are means ± SEM. (TIF) [file pone.0042803.s004.tif]

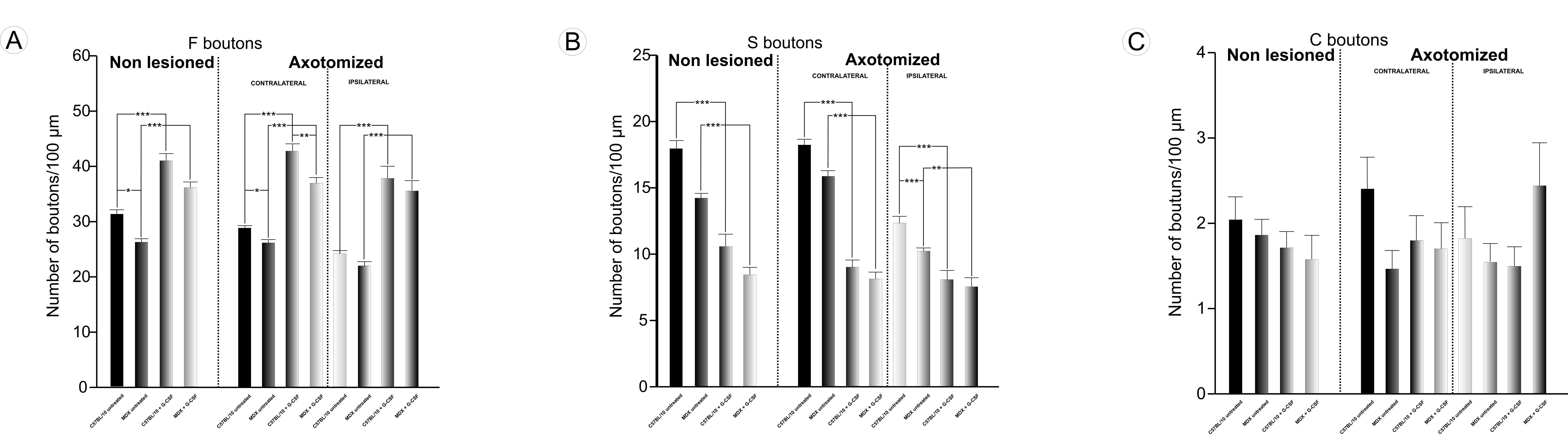

Supplement: Figure S5 — Synapse quantitative analysis. Representation of the ultrastructural quantitative analysis of the number of presynaptic terminals in neuronal membrane apposition/100 µm. (A) quantitative analysis of the number of F terminals in neuronal membrane apposition/100 µm. (B) quantitative analysis of the number of S terminals in neuronal membrane apposition/100 µm. (C) quantitative analysis of the number of C terminals in neuronal membrane apposition/100 µm. In all experiments: n = 5. In A, *p<0,05, **p<0,01, ***p<0.001 vs. CT. In B, **p<0.01, ***p<0.001 vs. CT, values are means ± SEM. (TIF) [file pone.0042803.s005.tif]
