# Supplementary material for: Granulocyte-Colony Stimulating Factor Improves MDX Mouse Response to Peripheral Nerve Injury
Source: PLoS One. 2012 Aug 13;7(8):e42803. doi: 10.1371/journal.pone.0042803 (PMC3418329; doi:10.1371/journal.pone.0042803)
Supplement: Table S1 — MHC I and synaptophysin immunolabeling quantification in non lesioned, untreated, placebo and treated with G-CSF groups. The data represent the mean value of the integrated density of pixels measured ± SEM. The different letters in each column represent the significant differences among the experimental groups. (DOCX) [file pone.0042803.s007.docx]

|  | MCH I | | Synaptophysin | |
| --- | --- | --- | --- | --- |
| GROUPS | **MDX** | **C57BL/10** | **MDX** | **C57BL/10** |
| Non lesioned untreated | – | – | 7.25 ± 0.23  **a** | 9.11 ± 0.18  **b** |
| Non lesioned + G-CSF | – | – | 13.05 ± 0.04  **c** | 13.95 ± 0.08  **d** |
| Contralateral untreated | – | – | 6.78 ± 0.08  **e** | 9.91 ± 0.10  **f** |
| Ipsilateral untreated | 17.45 ± 0.25  **A** | 24.46 ± 0.26  **B** | 4.80 ± 0.05  **g** | 6.67 ± 0.14  **h** |
| Contralateral + placebo | – | – | 6.98 ± 0.07  **e** | 9.50 ± 0.05  **f** |
| Ipsilateral+ placebo | 16.46 ± 0.26  **A** | 23.46 ± 0.25  **B** | 4.89 ± 0.08  **g** | 6.77 ± 0.11  **h** |
| Contralateral + G-CSF | – | – | 12.20 ± 0.21  **i** | 14.30 ± 0.10  **j** |
| Ipsilateral + G-CSF | 21.67 ± 0.21  **C** | 27.16 ± 0.23  **D** | 9.21 ± 0.37  **l** | 10.50 ± 0.10  **m** |
| *Ratios* |  |  |  |  |
| Axotomized / untreated | – | – | 0.71 ± 0.01  **n** | 0.69 ± 0.01  **n** |
| Axotomized / placebo | – | – | 0.70 ± 0.01  **n** | 0.71 ± 0.01  **n** |
| Axotomized / G-CSF |  |  | 0.73 ± 0.01  **n** | 0.73 ± 0.01  **n** |
