# Supplementary material for: Granulocyte-Colony Stimulating Factor Improves MDX Mouse Response to Peripheral Nerve Injury
Source: PLoS One. 2012 Aug 13;7(8):e42803. doi: 10.1371/journal.pone.0042803 (PMC3418329; doi:10.1371/journal.pone.0042803)
Supplement: Table S2 — GFAP I and IBA 1 immunolabeling quantification in non lesioned, untreated, placebo and treated with G-CSF groups. The data represent the mean value of the integrated density of pixels measured ± SEM. The different letters in each column represent the significant differences among the experimental groups. (DOCX) [file pone.0042803.s008.docx]

|  | GFAP | | IBA 1 | |
| --- | --- | --- | --- | --- |
| GROUPS | **MDX** | **C57BL/10** | **MDX** | **C57BL/10** |
| Non lesioned untreated | 7.10 ± 0.35  A | 4.74 ± 0.22  B | 5.48 ± 0.02  a | 5.36 ± 0.02  a |
| Non lesioned + G-CSF | 15.64 ± 0.04  C | 9.53 ± 0.25  D | 4.85 ± 0.07  a | 4.80 ± 0.03  a |
| Contralateral untreated | 11.45 ± 0.39  E | 8.58 ± 0.10  F | 4.59 ± 0.05  a | 4.25 ± 0.03  a |
| Ipsilateral untreated | 19.19 ± 0.22  G | 15.41 ± 0.42  H | 18.36 ± 0.20  b | 16.84 ± 0.14  b |
| Contralateral + placebo | 10.64 ± 0.16  E | 8.21 ± 0.11  F | 4.62 ± 0.06  a | 4.00 ± 0.05  a |
| Ipsilateral+ placebo | 18.02 ± 0.32  G | 13.73 ± 0.31  H | 18.63 ± 0.11  b | 17.32 ± 0.10  b |
| Contralateral + G-CSF | 16.34 ± 0.36  I | 13.25 ± 0.37  H | 5.51 ± 0.17  c | 5.23 ± 0.32  c |
| Ipsilateral + G-CSF | 28.65 ± 0.26  J | 21.95 ± 0.10  L | 20.94 ± 0.17  d | 19.73 ± 0.11  d |
| *Ratios* |  |  |  |  |
| Axotomized / untreated | 1.74 ± 0.05  M | 1.77 ± 0.04  M | 4.00 ± 0.02  e | 3.96 ± 0.05  e |
| Axotomized / placebo | 1.68 ± 0.02  M | 1.68 ± 0.01  M | 4.05 ± 0.04  e | 4.24 ± 0.14  e |
| Axotomized / G-CSF | 1.76 ± 0.03  M | 1.66 ± 0.05  M | 3.80 ± 0.12  e | 3.80 ± 0.24  e |
