# Supplementary material for: Granulocyte-Colony Stimulating Factor Improves MDX Mouse Response to Peripheral Nerve Injury
Source: PLoS One. 2012 Aug 13;7(8):e42803. doi: 10.1371/journal.pone.0042803 (PMC3418329; doi:10.1371/journal.pone.0042803)
Supplement: Table S3 — Transmission electron microscopy quantification for the percentage of covering and number of boutons/100 µm in non lesioned, untreated, placebo and treated with G-CSF groups. The data represent the mean value of the percentage covering and the number of boutons/100 µm ± SEM. The different letters in each column represent the significant differences among the experimental groups. (DOCX) [file pone.0042803.s009.docx]

|  | % Covering | | Number of boutons/100μm | |
| --- | --- | --- | --- | --- |
| GROUPS | **MDX** | **C57BL/10** | **MDX** | **C57BL/10** |
| Non lesioned untreated | 39.94 ± 1.56  **A** | 52.80 ± 1.43  **B** | 42.37 ± 2.04  **a** | 50.76 ± 0.85  **b** |
| Non lesioned + G-CSF | 49.50 ± 1.78  **C** | 60.4 ± 1.82  **D** | 42.76 ± 1.40  **a** | 53.30 ± 1.21  **b** |
| Contralateral untreated | 39.95 ± 1.10  **A** | 53.83 ± 0.75  **B** | 45.61 ± 0.73  **a** | 49.52 ± 0.42  **b** |
| Ipsilateral untreated | 33.62 ± 2.10  **E** | 40.84 ± 0.47  **D** | 32.21 ± 2.94  **c** | 39.57 ± 0.55  **d** |
| Contralateral + G-CSF | 54.94 ± 1.90  **C** | 62.47 ± 1.82  **D** | 46.32 ± 0.91  **a** | 54.10 ± 0.93  **b** |
| Ipsilateral + G-CSF | 47.34 ± 2.10  **F** | 52.90 ± 0.70  **G** | 43.73 ± 1.36  **a** | 49.88 ± 1.90  **b** |
