# Supplementary material for: Granulocyte-Colony Stimulating Factor Improves MDX Mouse Response to Peripheral Nerve Injury
Source: PLoS One. 2012 Aug 13;7(8):e42803. doi: 10.1371/journal.pone.0042803 (PMC3418329; doi:10.1371/journal.pone.0042803)
Supplement: Table S4 — Transmission electron microscopy quantification for the percentage of F, S and C boutons’ covering in non lesioned, untreated, placebo and treated with G-CSF groups. The data represent the mean value of the percentage covering ± SEM. The different letters in each column represent the significant differences among the experimental groups. (DOCX) [file pone.0042803.s010.docx]

| % Covering | | | | | | |
| --- | --- | --- | --- | --- | --- | --- |
|  | **F terminals** | | **S terminals** | | **C terminals** | |
| GROUPS | **MDX** | **C57BL/10** | **MDX** | **C57BL/10** | **MDX** | **C57BL/10** |
| Non lesioned untreated | 25.05 ± 0.85  A | 31.44 ± 0.97  B | 13.44 ± 0.86  a | 17.30 ± 0.43  b | 4.86 ± 0.71  *a* | 3.81 ± 0.65  *a* |
| Non lesioned + G-CSF | 37.73 ± 1.45  C | 44.79 ± 1.14  D | 8.04 ± 0.55  c | 12.00 ± 1.28  a | 3.72 ± 0.67  *a* | 3.58 ± 0.25  *a* |
| Contralateral untreated | 24.45 ± 1.25  A | 31.12 ± 1.19  B | 14.59 ± 0.63  a | 16.90 ± 0.57  b | 3.20 ± 0.42  *a* | 5.88 ± 0.65  *a* |
| Ipsilateral untreated | 20.60 ± 0.86  E | 25.00 ± 0.85  F | 8.46 ± 0.76  c | 14.45 ± 0.52  a | 3.43 ± 0.25  *a* | 5.72 ± 0.26  *a* |
| Contralateral + G-CSF | 42.92 ± 1.77  D | 48.68 ± 1.79  G | 7.34 ± 0.50  c | 10.12 ± 0.81  d | 4.68 ± 0.69  *a* | 3.67 ± 0.55  *a* |
| Ipsilateral + G-CSF | 34.63 ± 1.45  H | 40.74 ± 1.05  I | 7.53 ± 0.54  c | 6.98 ± 0.48  c | 5.18 ± 1.17  *a* | 4.17 ± 0.90  *a* |
