# Supplementary material for: Granulocyte-Colony Stimulating Factor Improves MDX Mouse Response to Peripheral Nerve Injury
Source: PLoS One. 2012 Aug 13;7(8):e42803. doi: 10.1371/journal.pone.0042803 (PMC3418329; doi:10.1371/journal.pone.0042803)
Supplement: Table S5 — Transmission electron microscopy quantification for the number of boutons/100 µm of F, S and C boutons’ covering in non lesioned, untreated, placebo and treated with G-CSF groups. The data represent the mean value of the number of boutons/100 µm± SEM. The different letters in each column represent the significant differences among the experimental groups. (DOCX) [file pone.0042803.s011.docx]

| Number of boutons/100μm | | | | | | |
| --- | --- | --- | --- | --- | --- | --- |
|  | **F terminals** | | **S terminals** | | **C terminals** | |
| GROUPS | **MDX** | **C57BL/10** | **MDX** | **C57BL/10** | **MDX** | **C57BL/10** |
| Non lesioned untreated | 26.36 ± 0.51  A | 31.78 ± 0.64  B | 14.69 ± 0.32  a | 16.92 ± 0.85  a | 1.90 ± 0.25  *a* | 2.05 ± 0.23  *a* |
| Non lesioned + G-CSF | 36.08 ± 1.02  C | 40.96 ± 1.11  C | 8.47 ± 0.56  b | 10.61 ± 1.09  b | 1.59 ± 0.33  *a* | 1.71 ± 0.19  *a* |
| Contralateral untreated | 26.24 ± 0.47  A | 29.52 ± 0.40  B | 15.69 ± 0.49  a | 17.14 ± 0.53  a | 1.51 ± 0.24  *a* | 2.34 ± 0.27  *a* |
| Ipsilateral untreated | 21.20 ± 0.43  D | 24.70 ± 0.61  E | 10.21 ± 0.30  c | 14.47 ± 0.41  d | 1.67 ± 0.26  *a* | 1.83 ± 0.31  *a* |
| Contralateral + G-CSF | 36.91 ± 1.01  C | 42.83 ± 1.22  D | 8.16 ± 0.51  b | 9.37 ± 0.55  b | 1.84 ± 0.31  *a* | 1.89 ± 0.40  *a* |
| Ipsilateral + G-CSF | 35.53 ± 1.80  C | 38.30 ± 2.18  D | 7.57 ± 0.67  e | 8.08 ± 0.69  e | 2.44 ± 0.50  *a* | 1.50 ± 0.23  *a* |
